# Direct synthesis of graphitic mesoporous carbon from green phenolic resins exposed to subsequent UV and IR laser irradiations

**DOI:** 10.1038/srep39617

**Published:** 2016-12-21

**Authors:** Mihai Sopronyi, Felix Sima, Cyril Vaulot, Luc Delmotte, Armel Bahouka, Camelia Matei Ghimbeu

**Affiliations:** 1 Lasers Department, National Institute for Lasers, Plasma and Radiation Physics, Atomistilor 409 bis, Magurele, Romania; 2 University of Bucharest, Faculty of Physics, Atomistilor 405, Magurele, Romania; 3 Université de Strasbourg, Université de Haute Alsace, Institut de Science des Matériaux de Mulhouse, CNRS UMR 7361, 15 rue Jean Starcky, 68057 Mulhouse, France; 4 IREPA LASER, Pôle API Parc d’Innovation 67400 Illkirch, France

## Abstract

The design of mesoporous carbon materials with controlled textural and structural features by rapid, cost-effective and eco-friendly means is highly demanded for many fields of applications. We report herein on the fast and tailored synthesis of mesoporous carbon by UV and IR laser assisted irradiations of a solution consisting of green phenolic resins and surfactant agent. By tailoring the UV laser parameters such as energy, pulse repetition rate or exposure time carbon materials with different pore size, architecture and wall thickness were obtained. By increasing irradiation dose, the mesopore size diminishes in the favor of wall thickness while the morphology shifts from worm-like to an ordered hexagonal one. This was related to the intensification of phenolic resin cross-linking which induces the reduction of H-bonding with the template as highlighted by ^13^C and ^1^H NMR. In addition, mesoporous carbon with graphitic structure was obtained by IR laser irradiation at room temperature and in very short time periods compared to the classical long thermal treatment at very high temperatures. Therefore, the carbon texture and structure can be tuned only by playing with laser parameters, without extra chemicals, as usually required.

Since being discovered^1^,^2^, mesoporous carbon (MC) has been attracted a lot of interest due to widespread application in various fields such as adsorption^3^,^4^, sensors^5^,^6^,^7^,^8^, energy storage and conversion^9^,^10^,^11^,^12^ or drug delivery systems^13^,^14^,^15^,^16^. The versatility is related to the outstanding properties such as high surface area, monodisperse mesopore size, tunable pore size and shape, chemical inertness and good electrical conductivity.

The first synthesis of MC was achieved through the so called hard-template approach^1^,^2^. A template made from a hard material (e.g. silica) possessing a mesoporous structure is filled with a carbon precursor (e.g. sucrose, furfuryl alcohol, propylene). The assembling is further carbonized, followed by a selective etching of the template which results in a mesoporous carbon material. Unfortunately, this method involves several steps, some of them unsafe (e.g. removal of the template in harsh acidic or basic conditions) and, in addition, is time consuming (taking up to several days)^17^.

To surpass these drawbacks, novel friendly environmental materials were used as carbon precursors and surfactants in the so called soft-template route^18^,^19^,^20^. The advantages of soft-template over hard-template consist of a faster synthesis, easiness, and several possible recipes between the precursors reflecting in various morphologies, structures and pore architectures. In such approach, the hard template is replaced by organic surfactant soft-templates which are able to self-assemble into defined nanospaces in the presence of carbon precursors (e.g. phenol/formaldehyde) *via* hydrogen bonding and/or covalent bonding in acidic or basic conditions. Such templates further allow organizing the carbon porosity in defined geometries but also to create the mesoporosity^21^.

In order to obtain the mesoporous carbon, the phenolic resin/template material undergoes a thermopolymerization at about 100 °C, followed by a carbonization step in inert atmosphere. This induces decomposition of the phenolic resin to carbon and, in the same time, decomposition of the template along with the formation of mesoporosity. Therefore, ordered honeycomb like, cubic like or worm-like pore networks may be obtained from the removed template micelles while their stability is kept by the thick pore walls and continuous interconnected framework^17^. During thermopolymerization which takes minimum of 12 hours, the cross-linking of phenolic resin is improving the rigidity that supports the thermal decomposition without altering the mesoporosity. In order to decrease the time of MC synthesis, several improvements were proposed by using high acidic conditions^18^ and/or high pressure (autoclaving)^22^ treatments. We have proposed recently a time efficient procedure to obtain mesoporous carbon *via* photopolymerization, i.e., light-assisted evaporation induced self-assembly (LA-EISA)^23^,^24^. We demonstrated that the classical long thermopolymerization at high temperatures (12 hours at 80 °C) may be replaced by 60 minutes of irradiation with an UV lamp source at room temperature. In addition, we have proved that the use of molecules capable to absorb light allowed to accelerate the reaction kinetics. The cross-linking and structure of the phenolic resins could be then significantly improved.

On the other hand, laser irradiation appeared to be a very appealing approach to synthesize and modify carbon structures. In a pioneering example, pure acetylene injected in a co-flow oxy-hydrogen flame was simultaneously irradiated by a CO_2_ laser covering whole acetylene flow. Hollow shell-shaped carbon nanoparticles with a high degree of crystallinity were then successfully synthesized by the laser assisted flame heating^25^. In another study, a Nd:YAG laser with 1064 nm wavelength was used to irradiate suspensions of black carbon in water. The laser energy absorption induced formation of hydrophilic groups of onion-like carbon surfaces with hollow cores^26^. Fluorescent carbon nanoparticles (CNPs) were synthesized by laser irradiation with a Nd:YAG (1064 nm) of graphite powders in different organic solvent suspensions^27^. Carbon-encapsulated magnetic nanoparticles were synthesized by laser irradiation with a pulsed Nd:YAG laser (355 nm) of a solution containing various metalocenes dissolved in xylene at room temperature and atmospheric pressure^28^. More recently, supported nanostructured carbons (porous carbons, graphenes, and carbon nanocomposites) prepared by CO_2_ laser has been reported as well^29^,^30^,^31^,^32^. The laser approach operates generally in air or inert atmosphere, at room temperature and provides localized high temperatures difficult to achieve by conventional thermal annealing, therefore, converting organic polymers into carbon materials in short time experiments.

However, the design of ordered mesoporous carbons with controlled textural and structural features by laser light along with the synthesis mechanism was not yet investigated. Herein, the influence of UV laser irradiation conditions (such as energy, repetition rate or exposure time) on the characteristics of phenolic resin and resulting carbon was investigated. The carbon organization, pore size and pore wall thickness could be thus tuned by irradiation conditions to get ordered carbon materials. In addition, we propose the CO_2_ laser irradiation as an alternative for classical thermal treatments as a one-step reaction to obtain graphitic porous carbons in very short times. Insights on the synthesis mechanism understanding to explain the texture and structural evolution with the laser parameters are proposed and supported by several analysis techniques.

## Results and Discussion

**Figure 1:**
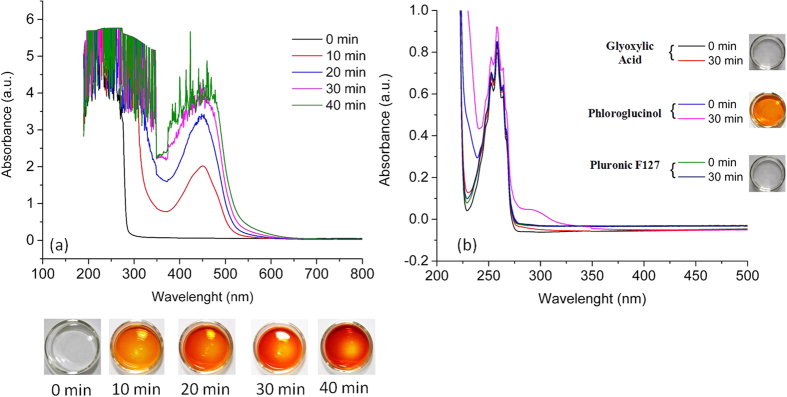
UV-VIS spectra of ethanolic solutions containing (**a**) all mixed precursors and (**b**) individual precursors, irradiated at 250mJ@18Hz between 0 and 40 min and the corresponding images of the solutions.

The carbon precursors (phloroglucinol-glyoxylic acid) and the template (Pluronic F127) were dissolved together in ethanol and irradiated at different time intervals. In order to observe and evaluate changes in the solution properties, the parameters such as energy per pulse (mJ), rate of repetition (number of pulses/second) and irradiation time (min) were monitored. A typical UV-VIS absorbance spectrum is provided in Fig. 1a and other supplementary spectra in Supporting Information (Figure S1). For all evaluated processing conditions, a peak around 450 nm appeares in the spectra. This is in line with the change of the solution coulour with irradiation dose from collorless to orange. When laser parameters such as energy, repetion rate and irradiation time increses, the irradiation dose increases. The reaction kinetics is faster and the color of solutions changes to dark orange and brown indicating more advanced polymerization reaction. This is accompanied by a gradually increase of the absorption peak at 450 nm.

To understand the evolution of solution with irradiation dose, the individual absorption of precursors was measured before and after irradiation. Each material was dissolved in EtOH and then irradiated under UV for 30 min. From plotted UV-VIS spectra in Fig. 1b, we observed that the glyoxylic acid and Pluronic F127 absorb in the UV regime (150 to 250 nm) but do not present any significant modifications after irradiation. This is confirmed by similar spectra overlap and unmodified solution color. We can also notice that phloroglucinol absorbs in the UV regime, in particular with predominance at 248 nm (laser wavelength), in line with the color change of solution from colorless to dark orange during laser irradiation. This modification may be due to the formation of phenoxyl type radicals when phloroglucinol is exposed to UV light, according to the following equation (Eq. 1)^33^. Hydroxyl HO∙ radicals can be formed as well under the UV light action on the ethanolic solution containing dissolved oxygen. Such reactive oxidant radicals may induce rapid photo-oxidation of phloroglucinol with the formation of quinone species (Eq. 1)^34^:


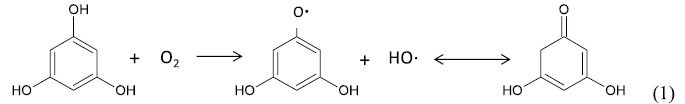


A detailed analysis of phloroglucinol was carried out in order to evaluate the evolution of irradiated solution at different time intervals. This changes the color after 5 minutes of irradiation only, meaning that the formation of radicals takes place rather fast. The formed phenoxyl radicals initiate and accelerate the reaction with the glyoxylic acid. This reaction induces the formation of new phenolic-resin based chromofore which absorbs in the VIS range region (450 nm), as seen in Fig. 1a. The self-assembly of phloroglucinol-glyoxylic acid phenolic resin *via* H-bonding with the PPO and PEO blocks of Pluronic template cannot be excluded either^23^.

After the irradiation process the samples are dried to obtain the phenolic-resin polymer which is further thermally treated. During the evaporation, the remaining ethanol is removed, the solution becomes more concentrated in Pluronic template which is then able to induce the self-assembly with the phenolic-resin *via* H-bonding interactions^35^. The as-obtained phenolic resin/template is heated at 600 °C to decompose the phenolic resin and the template with formation of porous carbon.

**Figure 2:**
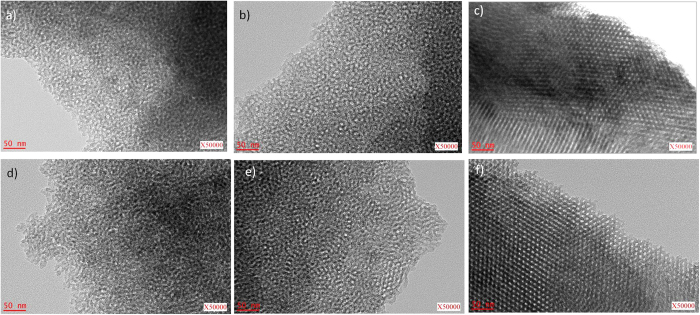
TEM images of mesoporous carbon obtained using different irradiation conditions of energy and repetition rate: (**a**) 160mJ@12Hz - 70 min, (**b**) 200mJ@12Hz - 70 min, (**c**)250mJ@12Hz - 70 min, (**d**) 250mJ@12Hz - 50 min, (**e**) 250mJ@15Hz - 50 min, (**f**) 250mJ@18Hz - 50 min.

TEM images of carbon materials synthesized by UV irradiation under different energies and repetition rates and further carbonized at 600 °C under argon are shown in Fig. 2.

At low energies, *i.e.*, 160 mJ (Fig. 2a) one can notice a worm-like morphology with both random oriented pores and some ordered pores. When increasing the energy to 200 mJ (Fig. 2b), the pores become more uniform, while at 250 mJ (Fig. 2c) the pores arrange in organized hexagonal structures. In Fig. 2d–f one can observe the same evolution of the pore arrangement by increasing the repetition rate instead of energy.

**Figure 3:**
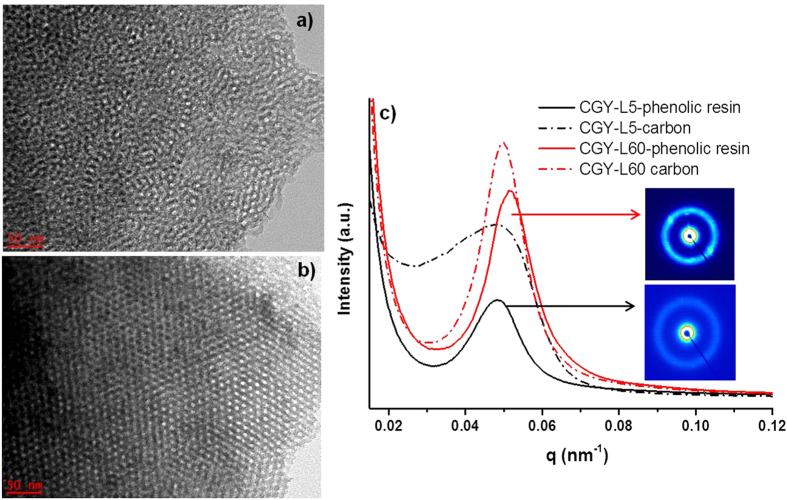
TEM of mesoporous carbon obtained by irradiation times of: (**a**) 5 min, (**b**) 60 min, (**c**) SAXS patterns of CGY-L5 and CGY-L60 phenolic resins and their derived carbons; in-set: 2D SAXS patterns (irradiation conditions: 250mJ@18Hz).

The time exposure was further tuned while keeping constant the energy and repetition rate at 250 mJ and 18 Hz, respectively (Fig. 3). With increase of exposure time from 5 to 60 min (Fig. 3a,b) the pores are more organized within the carbon network. In addition, parallel channels of carbon and the mesopores become well visible.

SEM analysis made on the same materials (Figure S2, Supporting Information) show that the materials is composed of particles with sizes of 50–200 μm for CGY-L5 and 50–100 μm for CGY-L60 (Figure S2a,d), respectively. The particles present a wrinkled morphology with a smooth surface where individual spherical particles can be noticed. For CGY-L5, the particles exhibit sizes comprised between 20 and 100 nm (Figure S2b,c) which tend to decrease for CGY-L60 to 20–50 nm but they become more abundant (Figure S2e,f).

To resume, by increasing the laser energy, repetition rate or irradiation time similar impact on the carbon pore organization can be induced. These results highlight the possibility of tuning the carbon pore size/architecture by laser parameters which, to our knowledge, was not reported before only by conventional chemical or thermal modification routes^17^,^21^,^36^.

The phenolic resins irradiated for 5 minutes and 60 minutes and their corresponding carbons have been analyzed by SAXS in order to get more information about the structural organization (Fig. 3c). For all materials, a peak located around 0.05 nm^−1^ (q) is noticed corresponding to (100) reflections of an ordered 2-D hexagonal structure^21^,^23^,^24^. When the irradiation time increases from 5 min (CGY-L5) to 60 min (CGY-L60), the peaks become more intense and narrow indicating improvement of phenolic resin and carbon organization, in good agreement with the TEM pictures (Fig. 3a,b). This can be also observed by the 2D-SAXS images showing well defined rings for material irradiated for 60 min (*in-set* Fig. 3c). We also notice that the peaks of the two carbons are placed rather in similar position. The lattice parameters, a_0,_ calculated for all materials are very similar and ranged between 14.0 and 14.6 nm (Table 1). It should be point it out that the determination of lattice parameter takes into account only the organized hexagonal domains which are able to induce the appearance of a SAXS peak. Therefore, for CGY-L5 which is characterized by coexisting worm-like and hexagonal morphologies an overall lattice parameter cannot be determined.

**Figure 4:**
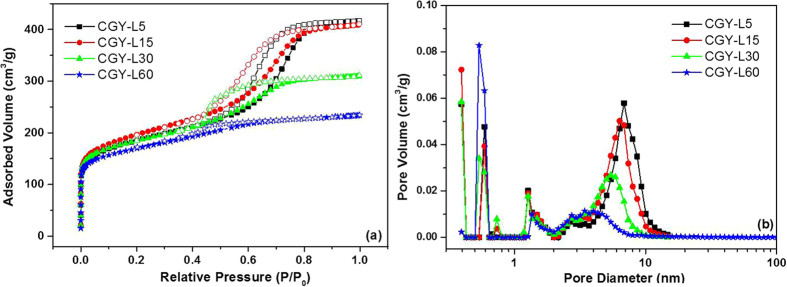
(**a**) Nitrogen adsorption/desorption isotherms of carbon materials obtained from precursor solutions irradiated by laser at different time intervals, (**b**) pore size distribution evaluated by NLDFT model; (irradiation conditions: 250mJ@18Hz).

In order to evaluate the influence of irradiation on the carbon textural properties (specific surface area, pore size and pore volume) the nitrogen adsorption/desorption isotherms were measured, as depicted in Fig. 4a. The isotherms present a specific type IV profile with a H1hysteresis loop, in agreement with other reports^21^,^37^. Such isotherm is characterized by an increase of the adsorbed nitrogen quantity in the low relative pressure range (P/P_0_ < 0.1) due to the micropores formed by the decomposition of phenolic-resin. The hysteresis is related to the capillary condensation in the mesopores formed by the thermal decomposition of Pluronic template^23^. The hysteresis loop for CGY-L5 (5 min irradiated) and CGY-L15 (15 min irradiated) is placed between 0.4 and 0.8 P/P_0_ and becomes much narrow in comparison with CGY-L30 (30 min irradiated) and CGY-L60 (60 min irradiated), 0.4–0.7 and 0.4–0.5 P/P_0_, respectively. This drop can be explained by a decrease in mesopore volume with irradiation time, as shown in Table 1. In same line, a shift towards smaller pores is noticeable in Fig. 4b. The average pore diameter for 5 minutes irradiated carbon (CGY-L5) is around 6.9 nm, while it decreases to 3.8 nm for 60 minutes irradiated carbon (CGY-L60).

Besides the shift towards smaller mesopores, the NLDFT pore size distribution shows a bimodal pore size distribution for all materials. The micropores (pore size < 2 nm) are also present which can be exploited in potential energy storage or adsorption applications^12^.

A clear trend of micropore size evolution with the irradiation time cannot be distinguished, for this reason CO_2_ adsorption was performed taking into consideration that CO_2_ is more suitable molecule for the determination of micropores smaller than 1 nm. Typical CO_2_ adsorption isotherms and pore size distribution for CGY-L5 and CGY-L60 are provided in Figure S3 (Supporting Information). The adsorption curves (Figure S3a) are almost overlapped and the determined microporous volumes are closed, i.e., 0.23 cm^3^∙g^−1^ for CGY-L5 and 0.21 cm^3^∙g^−1^ for CGY-L60, respectively, and comparable with those obtained by N_2_ adsorption (0.25 *vs.* 0.23 cm^3^∙g^−1^). The micropore size is centered on 0.5 nm for both materials (Figure S3b), few pores higher than 1.0 nm being visible in addition for CGY-L5.

To understand mesopore size evolution with the irradiation process, the wall thicknesses were calculated and the values are listed in Table 1. It can be clearly observed that the carbon wall thickness increases from 7.3 to 10.8 nm with the increase of the irradiation time from 5 min to 60 min. Taking into account that the lattice parameter is constant for hexagonal domains, it can be assumed that the decrease of the pore size with the irradiation time proceeds by the enlargement of the carbon wall. The synthesis of carbons with thick pore walls is of great interest since they provide higher mechanical and chemical stability. For instance, by hard-template is very difficult to modify the pore wall since they depend on the silica template features. By soft-template, the pore wall sizes are usually ranged between 4.0 and 7.0 nm due to the limitation of block copolymers characteristics. However, larger pores could be obtained mainly by the design of new templates^38^. Herein, we evidence the enlargement of pore wall thickness by irradiation approach which was not reported before. A possible mechanism for this will be discussed later in the manuscript.

The textural properties are collected in Table 1. All materials present rather high specific surface area: 623–706 m^2^∙g^−1^ and porous volumes ranged between 0.36–0.63 cm^3^∙g^−1^. The carbon textural values are similar to those obtained by classical EISA approach^21^,^37^. It is interesting to note that the irradiation time has no influence on the microporous volume which remains rather constant (Table 1) while the mesoporous volume significantly decreases. To understand this modification, it is worth to mention that the microporous volume is mainly derived from the decomposition/carbonization of phenolic resin while the mesopores are formed by the self-assembly and the decomposition of Pluronic^39^.

This suggests that the UV laser light influences the formation of micelles, their size, shape and spatial organization. On the other hand, the cross-linking of the phenolic resins under UV may modify their interactions with the Pluronic template.

The DSC and TGA analyses were employed to study the thermal behavior of phenolic resin irradiated for 5 minutes and 60 minutes (Figure S4, Supporting Information). For DSC spectra, one single peak is seen at around 100 °C, attributed to the removal of water due to the polycondensation reactions between the phloroglucinol and glyoxylic acid. We can observe that for CGY-L5 the peak is located at 101.7 °C, resulting in a heat (ΔH) of −127.5 J/g, while for the CGY-L60 the peak is centered at 111.7 °C corresponding to a heat of −84.4 J/g. Therefore, the heat cure of CGY-L60 is higher than the one of CGY-L5 suggesting more cross-linked phloroglucinol-glyoxylic acid phenolic resin with the increase of irradiation time. The TGA investigation (Figure S4b, Supporting Information) shows for both phenolic resins a total mass loss of approximately 80 wt.% at 700 °C. The constant weight loss from room temperature to 250 °C (~25 wt.%) corresponds to the removal of several species such as H_2_O, ethanol and fragments of hydrocarbons^40^. An important weight loss drop (~50 wt.%) is noticed between 250° to 400 °C, related to the decomposition of the triblock polymer Pluronic F127 with formation of water, COx, propyl, ethyl and methyl species^40^. At temperatures higher than 400 °C, the decomposition of the phenolic resin takes place. The derivative weight loss curve for CGY-L60 presents a narrow peak compared to CGY-L5, which may indicate less H-bonding between the phenolic resin and the micelles of surfactant, easy to be removed at 400 °C^21^,^40^.

**Figure 5:**
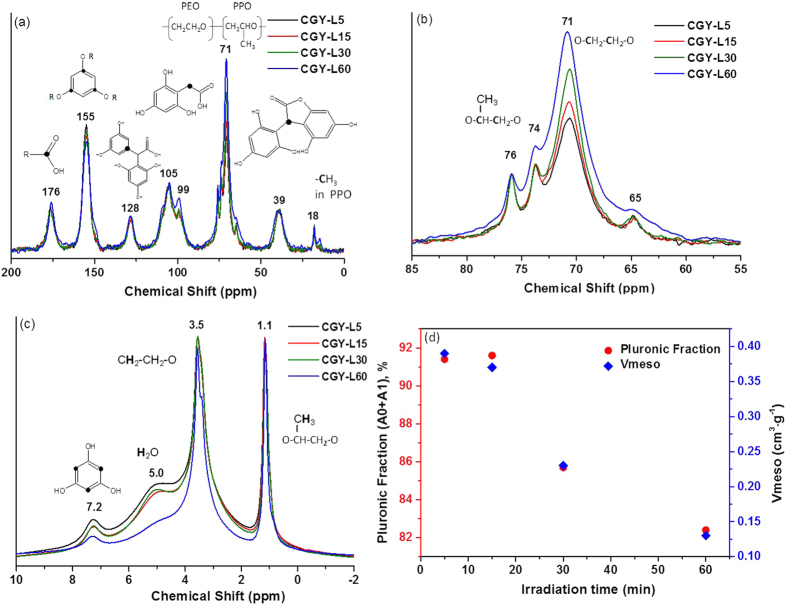
^13^C CP-MAS NMR (**a**,**b**) and ^1^H NMR (**c**) spectra of phenolic resins synthesized by irradiation at different times (irrdiations conditions:250mJ@18Hz); in-set: corresponding chemical structures and (**d**) relationship between the slightly mobile pluronic fraction and the mesoporous volume determined by ^1^H relaxation NMR.

To get further insights on the evolution of phenolic resins structure with the irradiation time, solid state ^13^C and ^1^H NMR was performed (Fig. 5). The ^13^C CP-MAS (cross-polarization magic angle spinning) spectra show several peaks placed in the same position for all materials (Fig. 5a).

The peaks observed at 176, 155, 128, 105, 99 and 39 *ppm* are assigned to the carbon atoms involved in the structure of phenolic resins formed by the polymerization reactions between the phloroglucinol and glyoxylic acid. These structures are reported in the *in-set* of the Fig. 5a and were described in detail in previous works^21^,^24^. The first peak (176 *ppm*) is related to carbon atoms involved in carboxylic function of glyoxylic acid or its derivates. The peaks at 155 *ppm and* 99 *ppm* correspond to unsaturated aromatic C bonded with OH and to the CH unsaturated bond respectively, in phloroglucinol. The peak at 128 ppm may be related to structures induced by photopolymerization. Phloroglucinol can form phenoxyl radicals (eq. 1) which react further with glyoxylic acid forming the structure labeled in the *in-set* of Fig. 5a.

The phloroglucinol and the glyoxylic acid may react by classical polymerization reactions giving rise to the so-called trihydroxy phenylacetic acid (structure corresponding to the peak placed at 105 *ppm*). Electrophilic aromatic substitution reactions occur between the trihydroxy phenylacetic acid and the phloroglucinol with the formation of carboxylic acid bridges. By subsequent condensation/elimination reactions new lactone bridges are formed (structure corresponding to peak 39 *ppm*).

The intense peak placed at ~70 *ppm* can be attributed to the carbon atoms bonded with O atoms (**C**H_2_-O-**C**H_2_) in the hydrophilic PEO moieties of Pluronic F127^41^. The other less intense proximity peaks at 74–76 *ppm* and at 18 *ppm* are corresponding to the carbons involved in ethyl (-**C**H-**C**H_2_) and methyl (-**C**H_3_) groups which belong to hydrophobic PPO moieties of Pluronic F127 template^42^.

A detail of spectrum around 70 *ppm* (Fig. 5b) allows observing peaks corresponding to the carbons in PPO (74 and 76 *ppm*), rather similar for all prepared phenolic resins using different time irradiation. On the contrary, the peak at 71 *ppm* assigned to the carbon in PEO (CH_2_-O-CH_2_) is increasing with the increase of the irradiation time. This may suggest the formation of other supplementary species with C-O-C groups. Either bridges between the trihydroxy phenylacetic acid and the glyoxylic acid or two trihydroxy phenylacetic acid structures are reasonable to be formed. This result suggests that the polymerization rate increases with the irradiation time in good agreement with TGA and DSC results.

Further insights on the chemical structure of these phenolic resins/template composites were assessed by ^1^H NMR (Fig. 5c). Several NMR signals are noticed at 7.2 *ppm*, 5.0 *ppm*, 3.5 *ppm* and 1.1 *ppm*, respectively. The peak at 7.2 *ppm* is assigned to H atoms of phloroglucinol aromatic ring while the one at 5.0 *ppm* belongs to the water or –OH groups. We observe that the intensity of these two peaks decreases with the increase of irradiation time. This suggests that the H atoms of phloroglucinol are substituted by condensation and water elimination reactions, as already seen by ^13^C NMR, confirming more advanced cross-linking of phenolic resin. The peaks at 3.5 *ppm* and 1.1 *ppm* correspond to protons in the ethylene oxides of PEO and propylene oxide of PPO moiety of Pluronic^42^. As noticed, the hydrophobic part of the template (PPO, 1.1 *ppm*) is not affected by the irradiation time since the intensity and the width of the peaks being kept constant. On the contrary, the peak corresponding to the hydrophilic PEO part (3.5 *ppm*) becomes narrower for longer irradiation times, indicating a higher mobility of the template. This result is in good agreement with the TGA and DSC analysis showing less interaction between the phenolic resin and the template with the increase of irradiation time.

To get more quantitative analysis about the mobile part of the system, i.e., Pluronic template, the ^1^H relaxation CPMG method was employed. This technique is very sensitive to mobile protons. Thus, rigid protons cannot be measured by this approach but they can be determined by extracting the quantity of mobile protons from the total theoretical quantity. Table 2 shows the spin-spin relaxation times, T2, of phenolic resins/template materials and their distribution obtained by mathematical treatment of relaxation curves (Figure S5, Supporting Information).

All materials are characterized by a bi-component system (Table 2). T2,1 values are ranging between 0.29 and 0.38 ms, significantly much lower compared to T2,2 (2.44 to 2.68 ms). This allow to associate T2,1 to the slightly mobile protons (A1) whereas the T2,2 to highly mobile pluronic template (A2). Both T2s tend to increase with the irradiation time, hence, the pluronic becomes more mobile. The pluronic slightly mobile fraction A1, is decreasing with the irradiation time (from 87.6% to 80.3%) in the favor of A2, i.e., highly mobile pluronic (12.4 to 19.7%). This means that irradiation plays an important role on the mobility of the template, which becomes more mobile with irradiation time, most probably due to weak interaction with the phenolic resin *via* H-bonding. This result is in well agreement with the TGA, DSC and ^1^H NMR results.

If we consider, that the theoretical composition of our reaction mixture contains 5,02 mol (n ^1^H) of pluronic template/100 g of material, the experimental quantities of Pluronic in materials are smaller (n ^1^H, Table 2). We observe that with the increase of irradiation time from 5 to 60 min, the quantity of protons in pluronic (n ^1^H) increases from 3.49 mmol to 4.49 mmol, approaching therefore the theoretical value.

This means that A1 and A2 fractions must be recalculated taking into consideration the real quantity of ^1^H protons observed in pluronic. For low irradiation times, one part of protons is not observed since is very rigid, well bonded to the phenolic resin. This explains the difference compared to the calculated values. In this case, if we express the fraction of less mobile protons (those there are still involved in self-assembly with the phenolic resin *via* H-bonding) as: (A0 + A1) (Table S1, Supporting Information) *vs.* the mesoporous volume, a good correlation will be found (Fig. 5d). The slightly mobile fraction of pluronic shows an almost linear relationship decrease with the irradiation time, accompanied by a linear decrease in the mesoporous volume. This means that when the irradiation proceeds, the H-bonding between the pluronic template and the phenolic resin is little by little suppressed. Thus, a part of Pluronic becomes very mobile, acting as “free” and probably is not anymore involved in the self-assembly which explains the decrease in the mesopore volume and size.

To understand these results, one can imagine that the pluronic chains may be in 3 possible states: under a high interaction within the cross-linked network of the phenolic resin (A0), under moderate interaction in the micelle of future mesopores (A1) and outside the system (A2). Then, a 3-steps process could be possible, in which under the evolution of the cross-linking under the irradiation time, the A0 fraction, is progressively pushed towards the pores (A1) whereas a similar amount is expelled from the system (A2). It is not yet clear if the observed small spherical particles on the surface of the carbons (SEM, Figure S2) are induced by this expulsion of Pluronic from the system.

**Figure 6:**
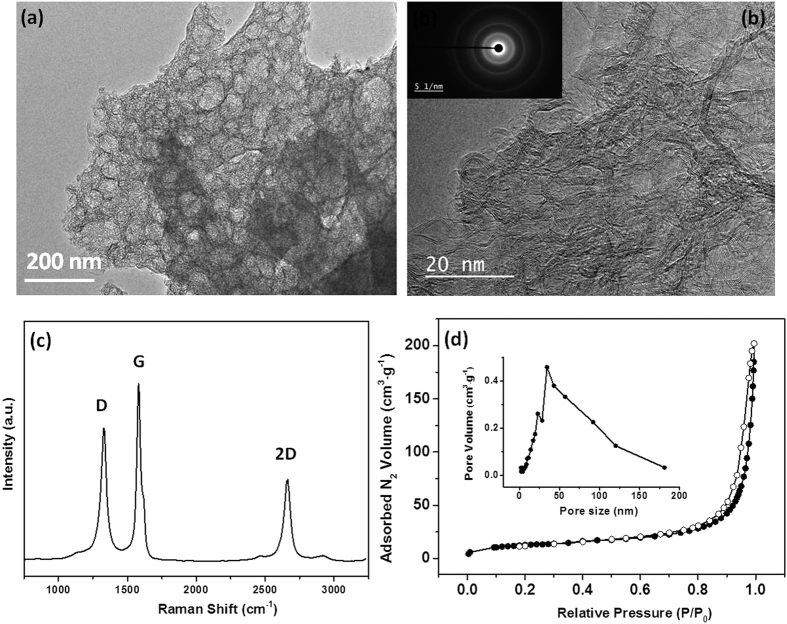
TEM pictures (**a**,**b**) of CGY-L60 irradiated by CO_2_ laser using different magnifications and corresponding SAED image (in-set); (**c**) Raman spectra (**d**) nitrogen adsorption/desorption isotherm and the BJH pore size distribution (in-set).

In addition, we have explored the possiblity of using an IR laser as an alternative to the thermal annealing classical procedure to carbonize the phenolic resin. With this aim, the phenolic resin irradiated by UV laser for 60 min (CGY-L60) was further irradiated in IR. TEM images, Raman spectra and the nitrogen adsorption/desorption isotherm of the obtained material are provided in Fig. 6.

The TEM pictures (Fig. 6a,b) reveal a porous morphology with pore size ranging between 20 and 100 nm. By HRTEM (Fig. 6b) it can be observed that the obtained material presents graphitic ribbons, which is highlighted as well by the *in-set* SAED diffraction image showing well visible concentric rings. This clearly demontrates the decomposition of phenolic resin and of template during the irradiation process and further graphitisation due to the high temperatures rechead during IR irradiations.

Raman spectroscopy was additionally performed (Fig. 6c) and three peaks are seen at 1334, 1583 and 2667 cm^−1^. The first two peaks are corresponding to the D band (defects) and G band (graphite) respectively, and their ratio gives indication about the graphitization level. The G band is more intense than D band demonstrating a graphitic structure. This is also corroborated by the presence of the third peak (2D) specific to graphitic carbon materials^43^. Under UV-irradiation only, the material does not show any specific peaks corresponding to carbon (Figure S6, Supporting Information) contrary to IR irradiation which efficently transforms the phenolic resin into graphitic carbon.

Such materials are of high interest in energy storage applications or others where high conductive materials are required. Besides, it should be pointed out that phenolic resins can be graphitised only at very high temperatures (>2500 °C) using classical thermal tratments^44^ or by using sacrificial transition metal catalysts^45^. It can be imagined that the local temperature induced by the CO_2_ laser is very high and eficently transform amorphous carbon into graphitised one as showed by Raman spectra. Therefore, the IR laser treatments present clear advantages compared to clasical methods.

The nitrogen adsorption/desorption isotherm of type IV is observed along with a hysteresis specific to mesopores (Fig. 6d). The size distribution of mesopores is broad compared to the thermal annealed carbons (CGY-L60) and ranged between 20 and 120 nm with a maximum centered at 50 nm (*in-set* Fig. 6d). This may be related to the fast phenolic resin/template carbonization under CO_2_ irradiation leading to the formation and release of CO_x_, H_2_O and H_2_ gaseous species and consequently to the pore expansion^31^. The surface area is 43 m^2^∙g^−1^ and the porous volume 0.11 cm^3^∙g^−1^, lower than the carbon materials obtained by UV (Table 1). Such decrease in the SSA is related to the graphitisation and densification of the material by removal of micropores.

The potential mechanism of the formation of mesoporous carbons under UV and IR can be explained as follows: during the UV irradiation, the Pluronic starts to self-assemble into micelles. The micelle maybe seen as a spherical core/shell structure, where the core is made by hydrophobic PPO and the shell is formed of PEO moieties. The micelles obtained at low irradiation times (GCY-L5) seems to have a rather worm-like shape as suggested by the obtained carbons with worm-like morphology (Fig. 3). As the irradiation proceeds (CGY-L30 and L60), the micelles are transformed into spherical shapes and self-assembled into hexagonal ordered assembly. It is possible that the size of the micelles is modified by irradiation as well, taking into consideration that the obtained carbons present smaller pore size. The modification of micelle size/shape in the presence of light has been already reported in several works^46^,^47^,^48^.

At the same time, phloroglucinol may form phenoxyl radicals under UV irradiation which further induce a better cross-linking of phloroglucinol with the glyoxylic acid. The –OH or –COOH groups of newly formed phenolic resin oligomer (phloroglucinol-glyoxylic acid) interact with the PEO segment of the Pluronic *via* hydrogen bonding^21^, forming a macromolecular assembly and eventually an ordered carbon. Therefore, when the irradiation time increases, the phenolic resin becomes better cross-linked as demonstrated by ^13^C and ^1^H NMR (Fig. 5) and DSC (Figure S4a). In the same time the H-bonding interactions are reduced as demonstrated by TGA results. Less OH or –COOH groups are then available to interact with the PEO fragments of the template.

During the thermal treatment, the decomposition of the phenolic resin and of the template occurs as demonstrated by TGA results (Figure S4b). With the increase of irradiation dose, the carbon organization changes from a worm-like to an ordered honeycomb like (TEM and SAXS, Fig. 3c). In addition, the pore size diminishes from 6.9 to 3.8 nm in the favor of carbon wall thickness which increases, while the overall cell size for organized domains is kept constant (Table 1). This organization may be accounted to the better cross-linking of the phenolic resin and formation of specific micelle organization under irradiation. ^1^H NMR relaxation studies (Fig. 5d) highlighted a relationship between the template mobility (H-bonding between the template and phenolic resin) and the mesoporous volume. When irradiation time is high, cross-linking under UV irradiation is induced and more template is not able anymore to establish H-bonding interactions with the phenolic resin. This is due to the reduced number of available -OH or -COOH groups. Corroborated with the modification of size and shape of the micelles during the UV irradiation, this leads to a decrease mesopore volume and size.

The carbonization step had also a great influence on the carbon porosity and structure. When performed under IR laser irradiation, the material graphitize due to the high local developed temperature. Moreover, the obtained material is porous (Fig. 6a,b) but differs from that obtained by classical thermal treatment (Fig. 3b). By thermal treatment, the heating proceeds very slowly (2 °C/min) to initially allow well cross-linking and to rigidify the phenolic resin before the removal of the template at around 400 °C. This is a required condition to obtain ordered mesoporous carbon by soft-template approach^49^.

In the case of IR irradiation, both the phenolic resin and the template are submitted to IR pulses. In this case the power density may reach tenths of thousandths of W/cm^2^ and the surface temperature several thousand degrees. At such high temperatures and rates of heating, chemical conversions of polymers in byproducts are induced^50^. The phenolic-resin polymer and the template polymer behave differently under the irradiation. The template possesses lower thermal stability and decomposes probably before complete phenolic resin carbonization, which may explain the loss of ordered porosity compared to classical thermal treatment. The graphitization of carbon under these conditions may be explained by the excessive and rapid raise of the temperature under irradiation which induces the decrease of specific surface area.

## Methods

### Material synthesis

For carbon synthesis, the following precursors were used: phloroglucinol (1,3,5 -benzenetriol, C_6_H_6_O_3_), glyoxylic acid monohydrate (C_2_H_2_O_3_·H_2_O), triblock copolymer Pluronic F127 (poly ethylene oxide)-*block-*poly (propylene oxide)-*block*-poly (ethylene oxide, PEO_106_PPO_70_PEO_106_, *M*_W_ = 12600 Da) and absolute ethanol (C_2_H_6_OH). The materials were purchased from Sigma-Aldrich and used without any additional modifications.

**Figure 7:**
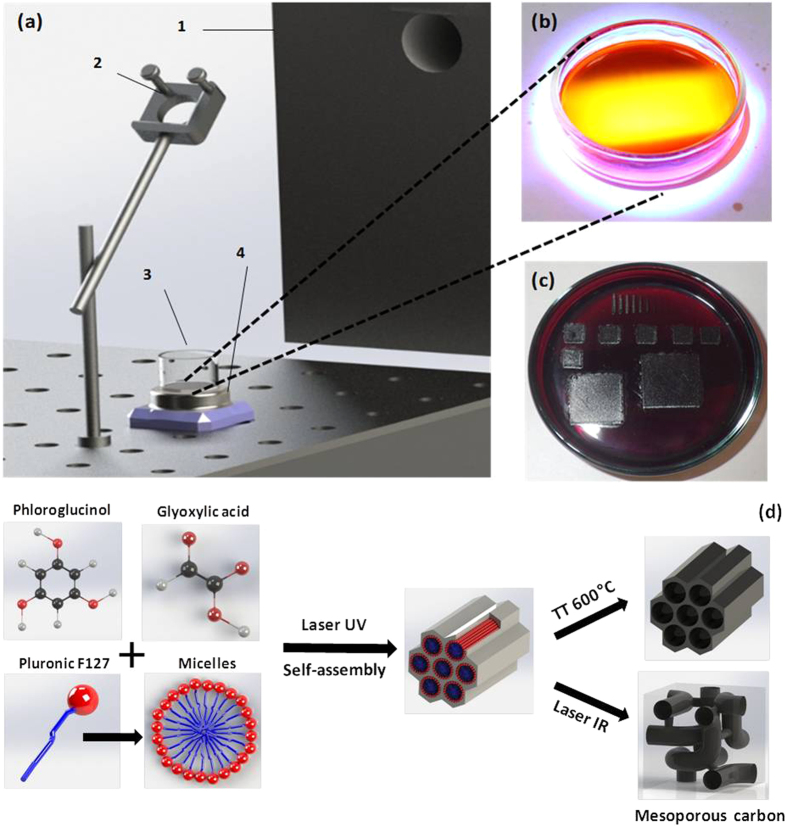
(**a**) Setup of the irradiation experiments: 1) laser source, 2) UV mirror, 3) petri dish with solution, 4) rotating system; (**b**) UV laser spot reflection on the liquid surface; (**c**) petri dish containing UV irradiated polymer and subsequently irradiated by IR laser; (**d**) schematic representation synthesis process.

In a first step, a solution was prepared as previously described^21^ by dissolving 0.82 g pholorglucionol, 0.61 g glyoxylic acid and 1.61 g Pluronic F127 in 40 ml of ethanol, in an Erlenmeyer flask wrapped in aluminum foil, by magnetic stirring for 60 min at 300 rpm at room temperature. Next, 10 ml of solution were disposed in Petri dishes and irradiated in air at room temperature with an excimer laser source (λ = 248 nm, pulse duration τ = 25 ns from Lambda Physik/Coherent, COMPEXPro 205) for time periods between 5 and 70 min. Energies within the range of 160 mJ–250 mJ and repetition rates from 12 Hz to 18 Hz were employed. The laser beam was deflected using a dielectric mirror for the UV regime (from Thorlabs) onto glass dishes containing the solution. To ensure a homogeneous irradiation of whole solution volume, the dish was rotated (Fig. 7a) and positioned at a certain distance from the mirror in order that unfocused laser spot would match the diameter of the dish. (Fig. 7b).

UV-VIS investigation was performed and photos of the solution were taken at specific time intervals between 2 and 70 minutes of UV laser irradiation. For UV-VIS analysis, a quantity of 0.3 ml was taken out from the dish and the spectra were recorded. The UV-VIS spectra were acquired on a double beam spectrophotometer (Cintra 10e, GBC, Scientific, Victoria, Australia) in a quartz cylinder of volume 3.5 (ml). For each spectra, the solution was diluted in EtOH, and measured in absorbance mode from 1200 nm to 190 nm with a scanning speed of 300 nm min^−1^ and a step size of 1 nm.

Afterwards, the solutions were dried overnight and carbonized. Two different routes were used to carbonize the obtained polymer: (i) thermal treatment at 600 °C (2 °C min^−1^) for 1 hour in inert atmosphere (Ar) and (ii) infrared CO_2_ laser using a DIAMOND 62 CO_2_ laser from Coherent ^®^ working at a wavelength of 10.6 μm and energy of 3 mJ. We tested several configurations with different power values, frequencies rates, scan speeds and spot sizes (that changes the power densities and the overlaps). The samples were placed under controlled atmosphere (Ar) and the irradiations operated at room temperature. The beam was focalized on the surface of the samples and lines were first patterned (upper part Fig. 7c) using different experimental parameters in order to find the most appropriate pyrolyse conditions. The color of the line and its homogeneity allow a fist appreciation of pyrolyse efficiency, i.e., darker lines indicating more advance carbonization process. To cover larger surfaces, the phenolic resins were irradiated with a scan speed of 30 mm/s, a power density of 2.4 kW/cm^2^, longitudinal overlap of 94%, pulse duration of 20 μs, frequency of 1 kHz and transverse overlap of 80%. The exposure time was 3.6 s for squares of 5 × 5 mm^2^.

The overall synthesis process is schematically represented in Fig. 7d.

### Material characterization

The small angle X-ray scattering (SAXS) investigations were carried out with a Rigaku Smax 3000 equipped with a rotating Cu anode Micromax-007HF (40 KV, 30 mA) and OSMIC CMF optics. The resulting carbon materials morphology and structure were investigated by TEM with a Philips M200 working at 200 kV. The textural properties of the carbon materials were determined with a Micromeritics ASAP 2020 instrument using a N_2_ adsorbate at −196 °C. The materials were degassed in vacuum at 300 °C for 12 h. The specific surface area (SSA) was obtained from the linear plot in the range of the relative pressure of 0.05–0.3 using the Brunauer-Emmett-Teller (BET) model. The microporous volume was calculated by using the equation of Dubinin-Radushkevich (DR) in the relative pressure region 10^−4^ to 10^−2^. The mesoporous volume was obtained by subtracting the micropore volume from the total pore volume of N_2_ adsorbed at the relative pressure of 0.95. The pore size distribution was determined by the N_2_ NLDFT model. The unit cell parameter, a_0_ was calculated using the formula 2·d_100_/√3 for hexagonal structures p6mm structures, where the d_100_ is the d-spacing of the (100) reflection. The thickness of the carbon wall (T_wall_) was calculated by subtracting the pore diameter from the lattice parameter (T_wall_ = a_0_ − D_pore_)^41^.

Cross-polarization (CP) ^13^C and ^1^H NMR spectra were acquired using a 90° 1 H pulse of 3.7 μs duration, a 1 [ms] contact time and a 5 s recycle delay. Free induction decays were acquired with a sweep width of 85 kHz. 8 K data points were collected over an acquisition time of 48 [ms]. All spectra were processed with a 24 to 40 Hz Lorentzian line broadening. Variable amplitude cross-polarization was used to minimize the intensity variations of the non-protonated aromatic carbons that are sensitive to Hartmann-Hahn mismatch at higher MAS (Magic Angle Spinning) rotation rates. Direct polarization (DP) with CW (continuous-wave) decoupling spectra represent the accumulation of 3000 scans and were acquired using a 45° 13 C pulse of 2.5 μs duration and a 20 s recycle delay. Chemical shifts were externally referenced to adamantane at 29.45 ppm. ^1^H NMR relaxation experiments were performed on a Bruker Minispec MQ-20 spectrometer. The dead-time of the receiver and the duration of the 90°and 180° pulses were 9 μs, 3.4 and 7.8 μs, respectively. The Carr Purcell Meiboom Gill (CPMG) sequence was used to measure spin-spin relaxation time *T*2 for the soft (mobile) domains exclusively. NMR signals were analyzed by using a discrete fitting method such as the Marquard t method (least-squares nonlinear regression technique). The used CPMG echo time was of 50 μs. A known amount of water was used as reference in order to quantify the mobile protons.

## Conclusions

In this work we successfully synthesized mesoporous carbon by laser assisted evaporation induced self-assembly under UV and IR irradiations. By pulsed UV laser irradiation of a solution consisting of friendly environmental carbon precursor and a template, the synthesis time could be reduced to 30 minutes as compared to couple of days by conventional EISA. In addition, by using appropriate processing conditions such as laser energy, pulse repetition rate or exposure time, the irradiation dose can be finely controlled. The irradiation conditions play an important role in the cross-linking of the phenolic resin but also in the self-assembly of phenolic resins with the template reflected in carbon materials with different textural and structural characteristics. The carbon organization, pore size and pore wall thickness were tuned by irradiation and ordered carbon materials could be obtained.

The mesoporous volume diminishes with the irradiation conditions while the morphology shifts from worm-like to ordered hexagonal. This could be explained by the increase of phenolic resin cross-linking which induces a decrease in the H-bonding with the pluronic template. This was quantitatively demonstrated by ^1^H relaxation NMR where, a linear relationship was found between the proton mobility in the template and the mesoporous volume.

The IR laser irradiation approach allowed reducing the overall synthesis process to a very short time periods, and obtaining porous carbon materials having different porosity with graphitic structure. Such synthesis approach may be extended to the design of microelectrodes for battery and supercapacitor for miniaturized devices.

## Additional Information

**How to cite this article**: Sopronyi, M. *et al*. Direct synthesis of graphitic mesoporous carbon from green phenolic resins exposed to subsequent UV and IR laser irradiations. *Sci. Rep.* **6**, 39617; doi: 10.1038/srep39617 (2016).

**Publisher's note:** Springer Nature remains neutral with regard to jurisdictional claims in published maps and institutional affiliations.

## Supplementary Material

Supplementary Information

## Figures and Tables

**Table 1:** Textural properties of carbon materials irradiated at different times and conditions (250mJ@18Hz).

Materials	SSA m^2^∙g^−1^	V_t_ cm^3^∙g^−1^	V_micro_ cm^3^∙g^−1^	V_meso_ cm^3^∙g^−1^	D_pore_ nm	a_0_ nm	Twall nm
CGY-L5	667	0.64	0.25	0.39	6.9	14.2	7.3
CGY-L15	706	0.63	0.26	0.37	6.6	14.2	7.6
CGY-L30	674	0.48	0.25	0.23	5.5	14.0	8.5
CGY-L60	623	0.36	0.23	0.13	3.8	14.6	10.8
CGY-L-IR	43	0.11	0.01	0.10	50	—	—

SSA – specific surface area, V_t_ – total pore volume, V_meso_ – mesopore volume, V_micro_ –micropore volume, D_pore_ – mesopore diameter calculated by NLDFT method; a_0_ – unit cell parameter calculated by SAXS, and T_wall_ – wall thickness.

**Table 2:** Relaxation times obtained by ^1^H NMR CPMG method of phenolic resins synthesized by irradiation at different times (irrdiations conditions: 250 mJ@18Hz).

Material	A1 (%)	T2,1 (ms)	A2 (%)	T2,2 (ms)	n ^1^H (mol/100 g)
**CGY-L5**	87.6	0.30	12.4	2.44	3.49
**CGY-L15**	86.8	0.29	13.2	2.44	3.18
**CGY-L30**	83.7	0.34	16.3	2.23	4.40
**CGY-L60**	80.3	0.38	19.7	2.68	4.49

Where: A1- Pluronic slightly mobile proton fraction; A2- Pluronic highly mobile proton fraction; n-number of mol of protons in the materials.
