# Supplementary material for: Direct synthesis of graphitic mesoporous carbon from green phenolic resins exposed to subsequent UV and IR laser irradiations
Source: Sci Rep. 2016 Dec 21;6:39617. doi: 10.1038/srep39617 (PMC5175163; doi:10.1038/srep39617)
Supplement: Supplementary Information [file srep39617-s1.doc]

**Direct synthesis of graphitic mesoporous carbon from green phenolic resins exposed to subsequent UV and IR laser irradiations**

Mihai Sopronyi^1,2^, Felix Sima^1^, Cyril Vaulot^3^, Luc Delmotte^3^, Armel Bahouka^4^, Camelia Matei Ghimbeu^3*^

1. Lasers Department, National Institute for Lasers, Plasma and Radiation Physics, Atomistilor 409 bis, Magurele, Romania

2. University of Bucharest, Faculty of Physics, Atomistilor 405, Magurele, Romania

3. Université de Strasbourg, Université de Haute Alsace, Institut de Science des Matériaux de Mulhouse, CNRS UMR 7361, 15 rue Jean Starcky, 68057 Mulhouse, France

4. IREPA LASER, Pôle API Parc d’Innovation 67400 Illkirch, France

* Corresponding author: [camelia.ghimbeu@uha.fr](mailto:camelia.ghimbeu@uha.fr)

| 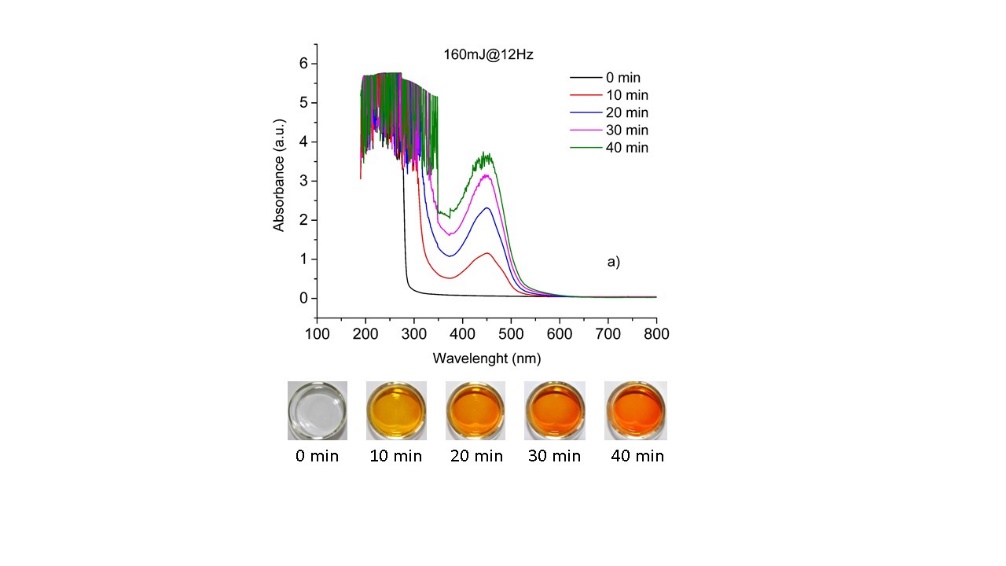 | 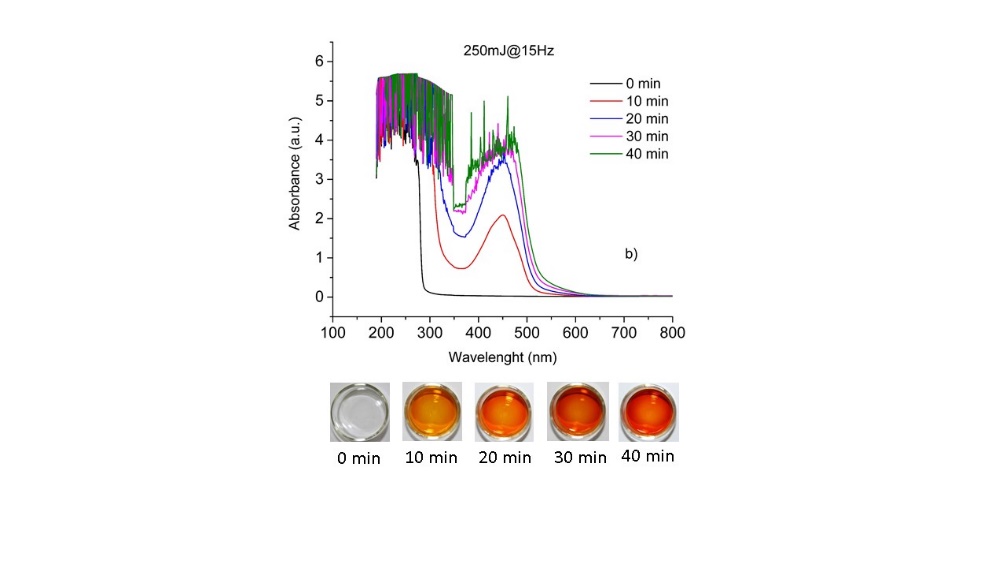 | 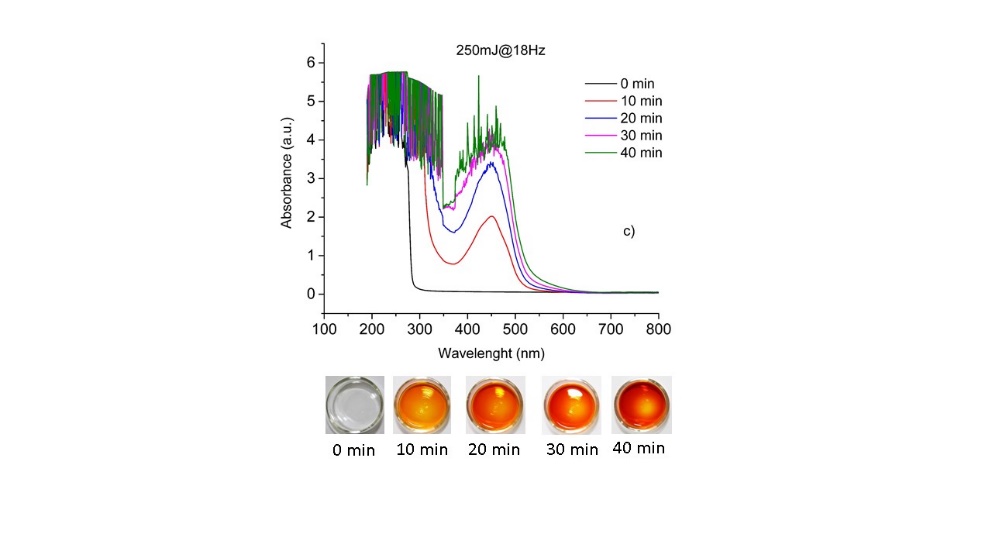 |
| --- | --- | --- |
| **Figure S1:** (up) UV-VIS spectra and photos of the solution irradiated under different conditions (a) 160 mJ@12Hz; (b) 250mJ@15Hz and (c)250 mJ@18Hz and different time intervals and (down) corresponding images of the irradiated solutions.  Scanning Electron Microscopy (SEM) analysis was made with a FEI Quanta 400 scanning electron microscope.    ***Figure S2:*** *SEM pictures of CGY-L5 (a-c) and CGY-L60 (d-f) materials.* | | |

The CO_2_ adsorption was performed at 273°C on a Micromeritics ASAP 2020 set-up. Prior to the analysis the materials were outgassed at 300°C under vacuum for one-night on the degassing port followed by 2h of out-gassing at the same temperature on the analysis port to remove the backfill gas. The pore size distribution was determined using the 2D-NLDFT model developed for carbon materials and implemented in SAIEUS (Micromeritics) [1,2].

***Figure S3:*** *CO_2_ adsorption isotherms and their corresponding 2D-NLDFT pore size distribution for CGY-L5 and CGY-L60 materials.*

For the thermo-gravimetric analysis (TGA), the materials were heated under air (10°C min^-1^) up to 800°C by using a Mettler-Toledo TGA 851e setup. Differential scanning calorimetry (DSC) was performed on a METTLER TOLEDO DSC 1, in an atmosphere of N_2_ with a debit of 100 ml/min, at a heating rate of 10K/min. The samples were investigated from -50°C up to 450°C.

|  |
| --- |
| ***Figure S4:*** *a) DSC and b) TGA of CGY-L5 and CGY-L60 phenolic resins (irradiation conditions: 250mJ@18Hz)* |

***Figure S5:*** *T2 relaxation curves* *obtained by ^1^H NMR CPMG method on phenolic resins prepared with different irradiation times*

**Table S1: Total measured quantities of ^1^H protons in the materials and their fractions**

| **Material** | **A0 (%)** | **A1 (%)** | **A2 (%)** |
| --- | --- | --- | --- |
| **CGY-L5** | 30.5 | 60.9 | 8.6 |
| **CGY-L15** | 36.7 | 54.9 | 8.4 |
| **CGY-L30** | 12.4 | 73.3 | 14.2 |
| **CGY-L60** | 10.6 | 71.6 | 17.6 |

*Where: A0 – rigid protons not measured by CPMG but calculated as follow: 100-A1-A2; A1- slightly mobile proton fraction and A2- highly mobile proton fraction measured by GPMG method;*

***Figure S6:*** *Raman spectra of phenolic resin (CGY-L60) irradiated under UV light for 60 min.*

Reference List

[1] Jagiello J, and Olivier J. 2D-NLDFT adsorption models for carbon slit-shaped pores with surface energetical heterogeneity and geometrical corrugation. Carbon 2013; 55: 70-80.

[2] Jagiello J, and Olivier J. Carbon slit pore model incorporating surface energetical heterogeneity and geometrical corrugation. Adsorption 2013; 19: 777-83.
